# Adaptation and Validation of the Italian Version of the Diabetes Self-Management Questionnaire (I-DSMQ) with an Additional Focus on Patients with Type 2 Diabetes

**DOI:** 10.3390/healthcare13050475

**Published:** 2025-02-21

**Authors:** Camilla Lombardo, Pierpaolo Vittorini, Leila Fabiani, Anna Rita Aleandri, Francesco Ciogli, Marta Fiorenza, Maria Scatigna, Assunta De Luca

**Affiliations:** 1 Department of Life, Health and Environmental Sciences, University of L’Aquila, 67100 L’Aquila, Italy; 2 Unit of Diabetology, San Camillo de Lellis Hospital, 02100 Rieti, Italy; 3 Medical Directorate, Local Health Authority-Sud East Tuscany, 52010 Chitignano, Italy

**Keywords:** diabetes, validity, reliability, cultural adaptation, DSMQ, self-management

## Abstract

**Background/Objectives:** The Diabetes Self-Management Questionnaire (DSMQ) provides a multidimensional measure of diabetes self-management behaviors essential for glycemic control. However, the DSMQ has never been validated in Italy despite the increasing prevalence of diabetes. This study aims to validate the Italian version of the DSMQ (I-DSMQ), with an additional focus on patients with type 2 diabetes. **Methods:** Two samples of patients attending the diabetology clinic at the ASL of Rieti were selected. The first sample included 70 patients diagnosed with either Type 1 Diabetes Mellitus (T1DM) or Type 2 Diabetes Mellitus (T2DM), while the second sample consisted of 99 individuals diagnosed with T2DM. Both groups completed the same questionnaire, the Italian version (I-DSMQ) of the original 16-item DSMQ, two times. **Results:** The results show that the Italian version maintains its reliability and consistency as an instrument applied to the Italian context in the self-management evaluation of patients with T1DM and T2DM. However, the Italian adaptation shows less satisfactory results considering only a subgroup of patients with T2DM. **Conclusions:** The I-DSMQ is a reliable and valid tool among Italian patients with T1DM and T2DM. Further insights are needed for the health usage domain if only T2DM patients are considered.

## 1. Introduction

According to the World Health Organization (WHO), about 422 million people worldwide have diabetes, with the majority living in low- and middle-income countries and 1.5 million deaths are directly attributed to diabetes each year [1]. The prevalence of diabetes has been steadily increasing over the past few decades [2]. According to data from the Italian Behavioural Risk Factor Surveillance System (PASSI), in Italy, slightly less than 5 percent of the adult population aged 18–69 years reported receiving a diabetes diagnosis in 2022–2023 [3]. The highest prevalences were found in some southern regions and among individuals over 50 years of age [4]. In fact, the prevalence of diabetics increases with age (it is 2% among adults younger than 50 years and approaches 9% among those aged 50–69 years) [3] and it is more common among men than women and in low-income populations due to lower educational attainment or socioeconomic status. Diabetes self-management is crucial for improving patients’ quality of life and for reducing health care costs. Indeed, self-care behaviors in chronic diseases have a positive and significant impact at all levels. In 2013, the Diabetes Self-Management Questionnaire (DSMQ) [5] was introduced to provide a multidimensional measure of diabetes self-management behaviors relevant to glycemic control in both major types of diabetes. A recent systematic review listed the DSMQ as one of only three diabetes self-management scales meeting the Consensus-based Standards for the Selection of Health Measurement Instruments (COSMIN) guidelines for reliable and recommended measurement tools [6]. Some specific aspects of the original DSMQ version were revised in the 27-item version of the instrument (DSMQ-R) to improve it [7]. The DSMQ is an undeniably valuable tool for investigating self-management in diabetic patients, and it is also already used in several countries [8,9]. DSMQ was designed to evaluate the answers of patients with both major diabetes types, type 1 (T1DM) and type 2 (T2DM). Moreover, T2DM is more frequent in older people and, according to the National Institute of Statistics (Istat), as of 2024, the Italian population older than 65 numbered 14.358 million and represented 24.3 percent of the total population. In addition, compared to 2023, the number of those over 80, the so-called oldest-old, has increased [10].

Another relevant aspect is that the COVID-19 pandemic has strained the Italian National Health Service (NHS), thus highlighting some critical issues in the infrastructure and organization. The pandemic has also underscored the need for more structured community medicine. The National Recovery and Resilience Plan (NRRP) and subsequent supporting national decrees were introduced in this context [11,12]. The aim is to ensure that all citizens have access to quality care [13,14] regardless of their geographical location through the organization of a new and more powerful community medicine that removes inequalities [15,16]. The availability of a validated tool to investigate self-management in diabetic patients, combined with the use of patient-reported outcome measures (PROMs) and patient-reported experience measures (PREMs), can represent a fundamental resource for both improving patient care and enhancing their awareness of care standards and appropriate care pathways. The Local Health Authority (ASL) in Rieti was committed to implementing and strengthening community medicine during and immediately after the pandemic. In fact, because of the predominantly hilly and mountainous territory surrounding Rieti, efficient community health care facilities are vital to ensure equal access to care for all citizens. In addition, the same ASL has invested in updating the care pathway dedicated to patients with diabetes.

In this context, this study aims to assess the validity, reliability, and relevance of the 16-item version of the DSMQ in measuring self-management among Italian diabetic patients.

We validated the 16-item version of the DSMQ questionnaire in Italian for several reasons. First, we aimed to ensure that the questionnaire could be completed within the timeframe of a diabetes consultation, avoiding excessively long completion times that might burden patients. Additionally, we prioritized a patient-centered approach, making the tool more accessible, particularly for older T2DM patients with lower health literacy and needing to complete the questionnaire independently without assistance. Then, the 16-item version is widely used in research and clinical settings, facilitating comparisons with previous studies and maintaining consistency with the existing literature.

Initially, this study focused on both types of diabetes. Then, given that patients with T2DM are, on average, not only older but also less educated and more distressed about self-management, we also chose to validate the DSMQ by exclusively considering these patients.

To the best of our knowledge, no prior research has evaluated the validity and reliability of the DSMQ in Italy. We believe it is crucial to have a tool in Italian to investigate self-management in diabetic patients primarily to improve the care process and further improve community medicine by detecting gaps to be filled based on what emerges from the questionnaires patients complete.

## 2. Materials and Methods

### 2.1. DSMQ Questionnaire

The DSMQ consists of 16 items categorized into four subscales: “Dietary Control” (DC) with four items, “Physical Activity” (PA) with three items, “Health-Care Use” (HU) with three items, and “Glucose Management” (GM) with five items [5]. Respondents rate how much each statement about their diabetes self-management behaviors applies to them using a four-point Likert scale. The DSMQ score is calculated as follows: first, all scores are summed after reversing the scores of nine negatively keyed items (items 5, 7, 10, 11, 12, 13, 14, 15, and 16 must be inverted). This sum is then transformed into a value ranging from 0 to 10, where 0 indicates the least effective self-care behavior and 10 indicates the most effective self-care behavior [5]. The questionnaire focuses on key aspects of diabetes self-management, addressing behaviors that directly influence glycemic control, such as adherence to dietary recommendations, regular physical activity, and appropriate health care utilization. The time frame for responses is the previous eight weeks, aligning with the time-dependent nature of HbA1c levels, ensuring the questionnaire reflects relevant self-care behaviors over a meaningful period. In the original study, the authors assessed the psychometric quality of the DSMQ by administering it, along with the Summary of Diabetes Self-Care Activities Measure (SDSCA), to 261 patients with type 1 or type 2 diabetes [5]. The DSMQ successfully differentiated between patients who were appropriately or inappropriately self-managing their diabetes, enabling the early identification of patients at high risk for poor diabetes outcomes [5].

### 2.2. Procedure

We organized the study as follows. First, we translated and culturally adapted the original DSMQ questionnaire from the British to the Italian context. The process consisted of two main activities:

- Translation process: this involved four stages—translation, synthesis, back-translation, and the acquisition of a consensus version following review by committee specialists.
- Assessment: this focused on evaluating the clarity, difficulty, and relevance of each item, as well as their psychometric properties.

In the first stage, a physician and a translator fluent in both English and Italian independently translated the DSMQ into Italian. One translator was familiar with the medical concepts underlying the items, while the other lacked any clinical background to ensure an unbiased linguistic perspective. The two translations were then synthesized into a single version, and any discrepancies were resolved by a neutral supervisor. The finalized Italian version was back-translated into English and subsequently reviewed by a committee of four experts that produced the final version, available as Attachment S1 in the Supplementary Materials. This version was then administered to a cohort of patients with both type 1 and type 2 diabetes to validate the I-DSMQ questionnaire. A test–retest procedure was also conducted to assess the stability of the results over time. We then repeated the same procedure with a second cohort of patients diagnosed exclusively with type 2 diabetes. This approach allowed us to explore the tool’s applicability in a population typically older and facing greater challenges in self-management and access to health care services.

### 2.3. Data Collection

The data used for the validation were collected from the Local Health Authority (ASL) in Rieti. This ASL was committed to implementing and strengthening community medicine during and immediately after the pandemic. In fact, because of the predominantly hilly and mountainous territory surrounding Rieti, efficient community health care facilities are vital to ensure equal access to care for all citizens. In addition, the same ASL has invested in updating the care pathway dedicated to patients with diabetes.

In early 2023, we invited patients with type 1 and type 2 diabetes to participate in the study. Seventy patients agreed, gave written consent, and compiled the questionnaire for the test (t_1_ = January 2023) and retest (t_2_ = March 2023) activities. We used these data to validate the I-DSMQ questionnaire.

In mid-2023, we focused on type 2 diabetes. A second cohort of 99 patients agreed, gave written consent, and compiled the questionnaire for the test (t_1_ = June/July 2023) and retest (t_2_ = December 2023) activities. We used these data to understand if the same questionnaire could be used for patients with type 2 diabetes and could be a valid representation of patients’ perspectives through self-reported measures. This transition to a second cohort was essential to determine which subscales required refinement to better suit the specific needs of these patients.

For both administrations, the inclusion criteria were:

- Age ≥ 18 years.
- Ability to understand the questions.
- Proficiency in the Italian language.
- The exclusion criteria were:
- Age < 18 years.
- Cognitive impairment, dementia, or other conditions that hinder self-management.

Incomplete questionnaires were excluded from the study.

Table 1 shows the demographics of the samples. Between the test and retest to validate the questionnaire for both major types of diabetes, there were some losses at follow-up that were not in the validation of the questionnaire for T2DM. This difference can likely be attributed to age-related factors and health care engagement patterns. Younger patients, predominantly those with T1DM, may have fewer diabetes-related complications and less frequent contact with medical services, resulting in higher attrition. Conversely, older patients with T2DM are typically included in regular checkup routines, making them easier to follow over a period of months, even in the absence of acute complications.

### 2.4. Statistical Analysis

The analyses were performed using R4.3.1 using the Lavaan 0.6-17 and psych 2.3.12 packages.

For descriptive statistics, means with standard deviations and frequency distributions were produced. We calculated skewness and kurtosis to verify normality. We considered data to be normally distributed if skewness was between −2 and +2 and kurtosis was between −7 and +7 [17].

We assessed the questionnaire’s reliability using Cronbach α (for the whole questionnaire and for each questionnaire’s subscale). We used the ranges proposed in [18] to provide a qualitative interpretation of the calculated alphas. We also used confirmatory factor analysis (CFA) and calculated several fit indices. As known, these indices measure how well data fit the hypothesized questionnaire structure and the related factors. The different indices use different strategies and assumptions to perform this measure. Therefore, we reported on the following three indices, which are among the most commonly used ones:

- Comparative fit index (CFI) [19]: it is an index that compares the model with the null model. The closer the CFI is to 1, the better the fit. A good fit is usually considered when the CFI > 0.90 [20].
- Root mean square error of approximation (RMSEA) [21]: measures the estimated discrepancy between the population and model-implied population covariance matrices per degree of freedom. The closer the RMSEA is to 0, the better the fit. The threshold of 0.08 [22] is usually considered for discriminating between a good and poor fit [21].
- Standardized root mean residual (SRMR): the index measures the difference between the observed and expected covariance. Similarly to the RMSEA, the closer the SRMR is to 0, the better the fit; the threshold of 0.08 discriminates between a good and poor fit [20,21].

Finally, for the test–retest analysis, we calculated the α at t_1_ and t_2_ and the R1F (reliability of average of all items for one time, i.e., random time effects) and RkF (reliability of average of all items and both times, i.e., fixed time effects).

## 3. Results

### 3.1. T1DM and T2DM

The descriptive statistics in Table S1, part of the Supplementary Materials, include the number of observations; the means and standard deviations; the normality skewness; and kurtosis coefficients. The values of all coefficients for all items suggest we can assume a normal distribution.

#### 3.1.1. Reliability of the Instrument

The internal consistency and reliability of the questionnaire, as well as of the four scale factors, were examined by the Cronbach’s α coefficient. For the whole questionnaire, we calculated an α = 0.90 (with 95% confidence intervals [0.87, 0.94]), which suggests the questionnaire to be reliable [18]. Table 2 details the reliability of each subscale and the reliability if an item is dropped. 

The reliability indices for the four subscales yielded values greater than 0.81, which suggests that the measures should be considered robust [18]. In connection with the correlations, they are located between 0.73 and 0.84 for Glucose Management (GM), between 0.79 and 0.87 for Dietary Control (DC), between 0.89 and 0.92 for Physical Activity (PA), and between 0.82 and 0.90 for Health Care Use (HU). In summary, all these results confirm that the instrument has satisfactory reliability.

#### 3.1.2. Confirmatory Factor Analysis

To verify the factorability of the data, we used the KMO index [23]. We calculated a value of 0.84 that, according to Kaiser’s guidelines [23], suggests data to be suitable for factor analysis. Thus, we used the Mardia’s test for multivariate normality. The findings indicated that the distribution was not normal (*p* < 0.001). Then, we used the Maximum Likelihood (ML) estimation approach to perform CFA. We achieved the following fit indices:

- CFI = 0.911;
- SRMR = 0.073;
- RMSEA = 0.096.

According to these indices, we can consider the fit satisfactory. Table 3 summarizes, for each item, its subscale, the descriptive statistics, skewness, kurtosis, and the factor loadings. The results show that the second-order confirmatory factorial analysis of the 15 items had good factorial loadings ranging from 0.62 to 0.91.

**Figure 1:**
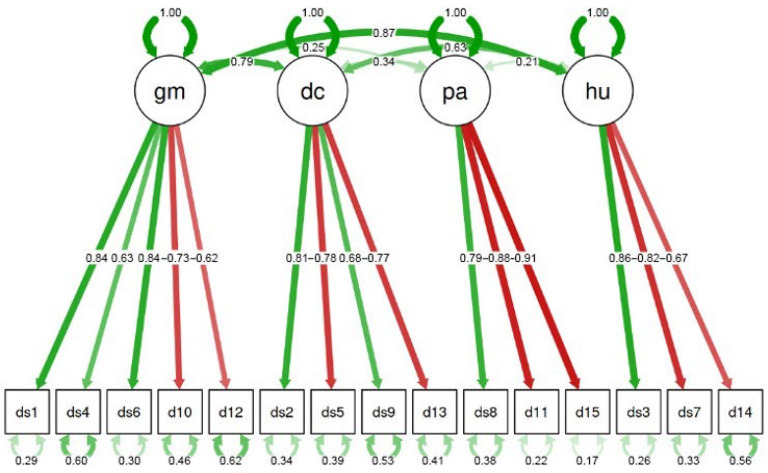
The final confirmatory analysis of I-DSMQ. Green arrows indicate positive correlations, while red arrows indicate negative ones.

Figure 1 graphically summarizes the above results. A green arrow suggests a positive correlation between the subscale and the related item, where, the stronger the color, the higher the correlation. A red arrow indicates a negative correlation and—similarly—the stronger the color, the higher the correlation. The numbers in the arrows are the corresponding loadings. The small arrows at the bottom of the figure represent the measurement errors or residual variances. These values indicate the portion of variance for each item that is not explained by the latent factor (or subscale) it is associated with. In other words, they show how much of the information contained in each item is explained by unobserved or random variables rather than by the latent factor. Lower values correspond to a better model fit.

In addition, the arrows on top of the figure show how the factors are related to each other. It is worth noting the high correlation existing only between GM and both DC (0.79) and HU (0.87), which suggests that glucose management is related to dietary control and access to health facilities.

#### 3.1.3. Test–Retest Analysis

The test–retest analysis returned the following results. The reliability at t_1_ was αt_1_ = 0.89 and equal to αt_2_ = 0.88 at t_2_. Moreover, we measured R1F = 0.97 and RkF = 0.99. These results imply very good stability of the results.

### 3.2. T2DM

The descriptive statistics in Table S2 (Supplementary Materials) include the number of observations, the means and standard deviations, the normality skewness, and kurtosis coefficients. The values of all coefficients suggest we can assume a normal distribution for all items.

#### 3.2.1. Reliability of the Instrument

The internal consistency and reliability of the questionnaire, as well as of the four scale factors, were examined by the Cronbach’s α coefficient. For the whole questionnaire, we calculated an α = 0.88 (with 95% confidence intervals [0.85, 0.92]), which suggests the questionnaire to be reliable [18]. Table 4 details the reliability of each subscale and the reliability if an item is dropped.

The reliability indices for the four subscales yielded mixed results. Glucose Management (GM), Dietary Control (DC), and Physical Activity (PA) have satisfactory results (i.e., 0.79, 0.84, and 0.76, respectively). On the other hand, Health Usage (HU) has a slightly acceptable result (i.e., 0.66). Questions 7 (I tend to avoid diabetes-related doctors’ appointments) and 14 (Regarding my diabetes care, I should see my medical practitioners more often) have the lowest correlation with the HU, with a correlation of 0.75 and 0.76, respectively. 

#### 3.2.2. Confirmatory Factor Analysis

Similarly to the above, we used the KMO index to verify the factorability of the data [23] (the value of 0.83 suggests that the data can be suitable for factor analysis), the Mardia’s test for multivariate normality (the distribution was not normal, *p* < 0.001), and the Maximum Likelihood (ML) estimation approach to perform CFA. We achieved the following fit indices:

- CFI = 0.816;
- SRMR = 0.095;
- RMSEA = 0.120.

According to these indices, we can consider the fit unsatisfactory, even if not too far from the common thresholds adopted to consider a CFA satisfactory. Table 5 summarizes, for each item, its subscale, the descriptive statistics, skewness, kurtosis, and the factor loadings. The results show that the second-order confirmatory factorial analysis of the 15 items had mixed results. We observe good factorial loadings ranging from 0.71 to 0.83 for DC and PA and slightly acceptable for GM and HU (with loadings ranging from 0.56 to 0.73).

#### 3.2.3. Test–Retest Analysis

The test–retest analysis returned the following results. The reliability at t_1_ was α_t1_ = 0.87 and equal to α_t2_ = 0.87 at t_2_. Moreover, we measured R1F = 0.97 and RkF = 0.98. These results—like those achieved for type 1 and type 2 diabetes—suggest very good stability of the results.

## 4. Discussion

This research investigated the psychometric characteristics of the Italian-language adaptation of the Diabetes Self-Management Questionnaire (DSMQ), abbreviated as I-DSMQ.

We performed two studies: the first involved patients with T1DM and T2DM, the actual target of the DSMQ questionnaire; the second involved only patients with T2DM to investigate the ability of the DSMQ instrument to focus only on this subgroup of patients, who, on average, are older and have co-morbidities and more difficulties in accessing primary care. In the first study, the cross-cultural adaptation and validation of the DSMQ in Italian demonstrated that the instrument maintains its validity and reliability within the Italian context. The translation and adaptation of the questionnaire were performed to ensure semantic and conceptual accuracy relative to the original version. Statistical analysis confirmed that the psychometric properties of the translated questionnaire were robust, employing various statistical techniques.

The questionnaire’s internal reliability was assessed by calculating Cronbach’s alpha, which showed an overall value of 0.90, indicating excellent internal consistency. Reliability analysis for specific subscales revealed alpha values ranging from 0.82 to 0.90, each reflecting good psychometric property.

Confirmatory factor analysis (CFA) was conducted to explore the factorial structure of the questionnaire. The CFA confirmed the factorial structure, with good fit indices. However, when focusing on type 2 diabetes patients, the analysis highlighted specific limitations in the Health Use (HU) domain. While questions 7 and 14 showed correlations of 0.75 and 0.76 with HU, their lower alpha value (0.66) compared to other subscales suggests a weaker internal consistency in this domain. These results indicate that the current items may not fully capture the complexity of health care usage among patients with T2DM. A more targeted approach, potentially incorporating additional or revised items specific to this population and the care model known to patients, could improve the reliability and validity of this subscale. For the type 2 diabetes cohort, the poorer fit indices in the CFA (CFI = 0.816, RMSEA = 0.120) further indicate challenges in adapting the DSMQ structure to this subgroup. These findings suggest that, while the DSMQ performs well overall, its application to patients with type 2 diabetes and who may have greater health care needs requires further refinement.

The motivation probably lies in the fact that the domain of HU refers to primary care and patients’ appropriate and effective use of primary care depends on several interconnected factors. Improving primary care visits requires targeted interventions that facilitate access, enhance trust in the system, and promote healthy and proactive behaviors. Many factors, like old age or low educational attainment, may hinder effective engagement with health care systems. Additionally, barriers such as limited access to digital tools and the attachment to traditional, outdated health care pathways can exacerbate these challenges. E-health systems and digitalization, paradoxically, may present greater challenges for elderly patients who are less familiar with digital tools. In this context, the consistent and trusting physician–patient relationship, along with standardized care models well introduced to patients, becomes even more crucial. This context underscores the importance of interventions aimed at strengthening patient–health care provider relationships, enhancing communication, and promoting health literacy through tailored education and support. This is also suggested by the high correlation discovered between the GC and HU subscales (see Figure 1).

GC, DC, and HU subscales are crucial in self-management but are profoundly influenced by knowledge and awareness of tailored care pathways and health literacy. Results suggest that even the best health care model fails if the patients do not have appropriate literacy tools to access and navigate it.

As crucial indicators of self-management ability, these domains collectively contribute to the overall quality of life. Overlooking the importance of self-management capacity by underestimating the role of knowledge and awareness regarding services tailored to one’s own condition risks undermining the effective use of health care resources. This issue is particularly accentuated in vulnerable populations, where achieving appropriate health care utilization is essential. More informed patients can deal better with the primary care system, i.e., with greater confidence and awareness [24]. In this case, the results of the HU domain for patients with T2DM are a warning sign that should not be ignored.

Moreover, the widespread presence of community health care facilities and primary care providers facilitates access to primary care services. In rural or mountainous areas, such as part of the Rieti ASL, accessibility and reduction in geographic barriers through the development of peripheral facilities and diabetes centers are crucial [25]. The Local Health Authority (ASL) of Rieti has developed a well-structured diagnostic–therapeutic care pathway (PDTA) for diabetic patients aimed at ensuring continuity of care and promoting a correct and co-ordinated use of primary care resources [26]. To face these challenges, systemic improvements such as better integration between levels of care, innovative organizational models, and targeted efforts to reduce economic barriers are essential. These measures would likely enhance the utility of the HU domain within the DSMQ for the Italian population with type 2 diabetes, making it a more robust tool for identifying gaps and improving self-management practices. 

### Limitations of the Study 

The main limitations of this study include the unknown literacy rate of the respondents, as well as their academic and socioeconomic level, which were not collected and could have influenced the results. In addition, the limited representativeness of the sample at the national level limits the generalizability of the results to the broader Italian population. Another aspect to consider is the enormous organizational heterogeneity found within the Italian health service between different ASLs, even from the same region; the organizational diversity of care pathways—in fact—makes it difficult to make valid hypotheses for the entire national territory regarding barriers in the use of services by citizens.

## 5. Conclusions

This study shows that the I-DSMQ is a reliable and valid tool in Italian patients with type 1 and 2 diabetes. However, further research is needed to assess diabetes self-management and its social determinants in a larger and more diverse Italian population. It should aim for more extensive and more varied samples to enhance generalizability and to better capture the variability in self-management behaviors across different demographic and socio-economic groups, also, by considering additional elements such as health literacy. In addition, the analysis showed that the instrument may not be sufficiently reliable when focusing only on patients with T2DM, particularly regarding self-management and health care use. Further research is needed to better set the instrument in measuring the health care utilization subscale for the subpopulation of patients with T2DM. In order to do this, a more detailed exploration of the impact of regional disparities in health care access on diabetes self-management is needed. Future research should investigate how differences in health care organization and service availability influence self-care behaviors and patient outcomes. Expanding the tool to account for these structural differences may enhance its validity in diverse health care settings.

## Figures and Tables

**Table 1:** Demographics of the study population.

Type 2				Type 1 and 2					Variable
Re-Test		Test		Re-Test		Test			
%	N	%	N	%	N	%	N		
	99		99		62		70		Sample size
49.5	49	49.5	49	43.5	27	44.3	31	Male	Gender
50.5	50	50.5	50	56.5	35	55.7	39	Female	
66.9 (7.9)		66.9 (7.9)		55.8 (16.6)		55 (16.4)		Mean (S.D.)	Age

**Table 2:** Internal consistency of the Italian version of Diabetes Self-Management Questionnaire (I-DSMQ).

Cronbach’s α [95% Confidence Intervals]	Cronbach’s α if the Item Is Dropped	Correlation	Items	Factors
0.84 [0.78, 0.90]	0.78	0.84	DSMQ 1	GM
	0.83	0.73	DSMQ 4	
	0.79	0.82	DSMQ 6	
	0.8	0.8	DSMQ 10 (−)	
	0.83	0.73	DSMQ 12 (−)	
0.86 [0.81, 0.91]	0.81	0.86	DSMQ 2	DC
	0.79	0.87	DSMQ 5 (−)	
	0.85	0.79	DSMQ 9	
	0.82	0.83	DSMQ 13 (−)	
0.90 [0.85, 0.94]	0.89	0.89	DSMQ 8	PA
	0.83	0.92	DSMQ 11 (−)	
	0.83	0.92	DSMQ 15 (−)	
0.82 [0.75, 0.90]	0.77	0.85	DSMQ 3	HU
	0.66	0.9	DSMQ 7 (−)	
	0.83	0.82	DSMQ 14 (−)	

**Table 3:** Descriptive statistics for each item.

Loading	Factor	Kurtosis	Skewness	St.Dev.	Mean	Variable
0.84	GM	−1.07	−0.32	0.91	1.99	DSMQ 1
0.63		−0.35	−0.86	0.63	2.5	DSMQ 4
0.84		−1.14	−0.14	0.98	1.76	DSMQ 6
−0.73		−1.27	−0.17	1.06	1.49	DSMQ 10 (−)
−0.62		0.8	0.44	0.93	1.06	DSMQ 12 (−)
0.81	DC	−1	0.05	0.94	1.63	DSMQ 2
−0.78		−0.75	0	0.85	1.34	DSMQ 5 (−)
0.68		−0.43	0.01	0.75	1.59	DSMQ 9
−0.77		−0.58	0.56	0.86	0.91	DSMQ 13 (−)
0.79	PA	−0.7	0.28	0.9	1.33	DSMQ 8
−0.88		−1.36	0.03	1.07	1.33	DSMQ 11 (−)
−0.91		−1.21	0	1	1.33	DSMQ 15 (−)
0.86	HU	−1	−0.46	0.95	2.03	DSMQ 3
−0.82		−0.56	0.74	0.93	0.84	DSMQ 7 (−)
−0.67		−1.04	−0.01	0.95	1.34	DSMQ 14 (−)

**Table 4:** Internal consistency of the Italian version of Diabetes Self-Management Questionnaire (I-DSMQ) for T2DM.

Cronbach’s α [95% Confidence Intervals]	Cronbach’s α if the Item Is Dropped	Correlation	Items	Factors
0.79 [0.73, 0.86]	0.8	0.65	DSMQ 1	GM
	0.75	0.76	DSMQ 4	
	0.74	0.78	DSMQ 6	
	0.75	0.78	DSMQ 10 (−)	
	0.76	0.75	DSMQ 12 (−)	
0.84 [0.79, 0.89]	0.82	0.8	DSMQ 2	DC
	0.78	0.86	DSMQ 5 (−)	
	0.81	0.82	DSMQ 9	
	0.81	0.82	DSMQ 13 (−)	
0.76 [0.68, 0.84]	0.74	0.82	DSMQ 8	PA
	0.68	0.84	DSMQ 11 (−)	
	0.69	0.84	DSMQ 15 (−)	
0.66 [0.55, 0.77]	0.5	0.8	DSMQ 3	HU
	0.61	0.75	DSMQ 7 (−)	
	0.59	0.76	DSMQ 14 (−)	

**Table 5:** Descriptive statistics for each item.

Loading	Factor	Kurtosis	Skewness	St.Dev.	Mean	Variable
0.64	GM	−1.45	−0.13	0.8	2.07	DSMQ 1
0.61		0.28	−0.86	0.78	2.26	DSMQ 4
0.67		−1.04	−0.03	0.77	1.94	DSMQ 6
−0.73		0.19	0.71	0.68	0.67	DSMQ 10 (−)
−0.68		−0.13	0.95	0.59	0.44	DSMQ 12 (−)
0.71	DC	−0.93	−0.13	0.93	1.7	DSMQ 2
−0.83		−0.3	0.63	0.83	0.89	DSMQ 5 (−)
0.73		−0.61	−0.27	0.81	1.9	DSMQ 9
−0.8		4.3	1.92	0.67	0.41	DSMQ 13 (−)
0.73	PA	−0.56	−0.26	0.84	1.77	DSMQ 8
−0.74		−0.64	0.18	0.59	0.71	DSMQ 11 (−)
−0.78		−0.51	0.07	0.58	0.75	DSMQ 15 (−)
0.73	HU	−1.1	−0.34	0.73	2.21	DSMQ 3
−0.56		−0.24	0.89	0.57	0.43	DSMQ 7 (−)
−0.64		−0.12	−0.06	0.55	0.83	DSMQ 14 (−)

## Data Availability

The datasets used and/or analyzed during the current study are available from the corresponding author upon reasonable request.

## Supplementary Materials

The following supporting information can be downloaded at: https://www.mdpi.com/article/10.3390/healthcare13050475/s1, Attachment S1: Italian Diabetes Self-Management Questionnaire (I-DSMQ); Table S1: Descriptive statistics of the T1DM–T2DM sample; Table S2: Descriptive statistics of the T2DM sample.
