# Supplementary material for: Adaptation and Validation of the Italian Version of the Diabetes Self-Management Questionnaire (I-DSMQ) with an Additional Focus on Patients with Type 2 Diabetes
Source: Healthcare (Basel). 2025 Feb 21;13(5):475. doi: 10.3390/healthcare13050475 (PMC11899450; doi:10.3390/healthcare13050475)
Supplement: Supplementary file 1 [file healthcare-13-00475-s001.zip › Attachment S1.pdf]

Italian Diabetes Self-Management Questionnaire (I-DSMQ)

Le seguenti affermazioni descrivono le attività di cura per il diabete autogestite e di automedicazione, come prendere farmaci, controllare la glicemia, ecc. Pensando alle cure autogestite/automedicazione nelle ultime 8 settimane, specifichi per favore quanto è d'accordo con ciascuna delle seguenti affermazioni.	Completamente d'accordo	D'accordo	Parzialmente d'accordo	In disaccordo
1. Controllo il livello della mia glicemia con cura e attenzione<br>☐ Il controllo della glicemia non è necessario per la mia terapia	☐	☐	☐	☐
2. Il cibo che scelgo rende facile raggiungere livelli soddisfacenti di glicemia	☐	☐	☐	☐
3. Rispetto tutti gli appuntamenti raccomandati dal medico per la cura del mio diabete	☐	☐	☐	☐
4. Prendo i farmaci per il diabete (ad es. Insulina, compresse) come prescritto<br>☐ Non è necessario che io prenda farmaci	☐	☐	☐	☐
5. A volte mangio molti dolci o cibi ricchi di carboidrati	☐	☐	☐	☐
6. Registro regolarmente i livelli della mia glicemia (o visualizzo i dati con il glucometro)<br>☐ Il controllo della glicemia non è necessario per la mia terapia	☐	☐	☐	☐
7. Tendo ad evitare gli appuntamenti per il diabete con il medico	☐	☐	☐	☐
8. Svolgo regolarmente attività fisica per raggiungere livelli soddisfacenti di glicemia	☐	☐	☐	☐
9. Seguo rigorosamente le raccomandazioni dietetiche fornitemi dal mio medico di famiglia o dal diabetologo	☐	☐	☐	☐
10. Non misuro abbastanza frequentemente la mia glicemia come sarebbe necessario per mantenere un buon controllo glicemico<br>☐ Il controllo della glicemia non è necessario per la mia terapia	☐	☐	☐	☐
11. Evito l'attività fisica anche se migliorerebbe il mio diabete	☐	☐	☐	☐
12. Salto l'assunzione dei miei farmaci per il diabete (ad es. Insulina, compresse) o tendo a dimenticare di prenderli<br>☐ Non è necessario che io prenda farmaci	☐	☐	☐	☐
13. A volte faccio delle vere e proprie abbuffate (non dovute all'ipoglicemia)	☐	☐	☐	☐
14. Per il mio diabete dovrei vedere i medici che mi seguono più spesso	☐	☐	☐	☐
15. Tendo a non fare l'attività fisica programmata	☐	☐	☐	☐
16. L'autogestione delle cure/automedicazione che riesco a praticare per il mio diabete è scarsa	☐	☐	☐	☐
