# Supplementary material for: Adaptation and Validation of the Italian Version of the Diabetes Self-Management Questionnaire (I-DSMQ) with an Additional Focus on Patients with Type 2 Diabetes
Source: Healthcare (Basel). 2025 Feb 21;13(5):475. doi: 10.3390/healthcare13050475 (PMC11899450; doi:10.3390/healthcare13050475)
Supplement: Supplementary file 1 [file healthcare-13-00475-s001.zip › Table S1.pdf]

Table S1. Descriptive statistics of the T1DM T2DM sample.

	Test					Re-Test				
	N	Mean	S.D.	Skew.	Kurt.	N	Mean	S.D.	Skew.	Kurt.
DSMQ 1	67	1.99	0.91	-0.32	-1.07	59	2.05	0.90	-0.38	-1.09
DSMQ 2	70	1.63	0.94	0.05	-1.00	62	1.68	0.84	0.01	-0.77
DSMQ 3	70	2.03	0.95	-0.46	-1.00	62	2.13	0.93	-0.61	-0.85
DSMQ 4	68	2.50	0.63	-0.86	-0.35	60	2.55	0.62	-1.02	-0.07
DSMQ 5 (-)	70	1.34	0.85	0.00	-0.75	62	1.21	0.79	0.21	-0.48
DSMQ 6	70	1.76	0.98	-0.14	-1.14	62	1.73	0.91	-0.09	-0.93
DSMQ 7 (-)	70	0.84	0.93	0.74	-0.56	62	0.84	0.96	0.86	-0.36
DSMQ 8	70	1.33	0.90	0.28	-0.70	62	1.29	0.89	0.36	-0.62
DSMQ 9	70	1.59	0.75	0.01	-0.43	62	1.63	0.79	-0.03	-0.54
DSMQ 10 (-)	67	1.49	1.06	-0.17	-1.27	60	1.33	1.02	-0.02	-1.27
DSMQ 11 (-)	70	1.33	1.07	0.03	-1.36	62	1.35	1.07	0.06	-1.32
DSMQ 12 (-)	68	1.06	0.93	0.44	-0.80	60	0.97	0.97	0.39	-1.25
DSMQ 13 (-)	70	0.91	0.86	0.56	-0.58	62	0.77	0.82	0.78	-0.15
DSMQ 14 (-)	70	1.34	0.95	-0.01	-1.04	62	1.15	0.90	0.25	-0.90
DSMQ 15 (-)	70	1.33	1.00	0.00	-1.21	62	1.34	1.05	0.05	-1.30
DSMQ 16	70	1.34	0.78	-0.11	-0.63	62	1.16	0.75	-0.03	-0.81
