# Supplementary material for: Adaptation and Validation of the Italian Version of the Diabetes Self-Management Questionnaire (I-DSMQ) with an Additional Focus on Patients with Type 2 Diabetes
Source: Healthcare (Basel). 2025 Feb 21;13(5):475. doi: 10.3390/healthcare13050475 (PMC11899450; doi:10.3390/healthcare13050475)
Supplement: Supplementary file 1 [file healthcare-13-00475-s001.zip › Table S2.pdf]

Table S2. Descriptive statistics of the T2DM sample.

	Test					Re-Test				
	N	Mean	S.D.	Skew.	Kurt.	N	Mean	S.D.	Skew.	Kurt.
DSMQ 1	95	2.07	0.80	-0.13	-1.45	95	2.11	0.76	-0.18	-1.29
DSMQ 2	99	1.70	0.93	-0.13	-0.93	99	1.80	0.94	-0.33	-0.81
DSMQ 3	99	2.21	0.73	-0.34	-1.10	99	2.20	0.73	-0.32	-1.09
DSMQ 4	97	2.26	0.78	-0.86	0.28	98	2.28	0.77	-0.77	-0.09
DSMQ 5 (-)	99	0.89	0.83	0.63	-0.30	99	0.88	0.85	0.72	-0.13
DSMQ 6	97	1.94	0.77	-0.03	-1.04	97	1.95	0.76	-0.20	-0.58
DSMQ 7 (-)	99	0.43	0.57	0.89	-0.24	99	0.49	0.64	0.92	-0.27
DSMQ 8	99	1.77	0.84	-0.26	-0.56	99	1.80	0.86	-0.47	-0.33
DSMQ 9	99	1.90	0.81	-0.27	-0.61	99	1.83	0.81	-0.26	-0.49
DSMQ 10 (-)	96	0.67	0.68	0.71	0.19	96	0.70	0.71	0.67	-0.17
DSMQ 11 (-)	99	0.71	0.59	0.18	-0.64	99	0.70	0.66	0.62	0.22
DSMQ 12 (-)	97	0.44	0.59	0.95	-0.13	97	0.44	0.61	1.02	-0.02
DSMQ 13 (-)	99	0.41	0.67	1.92	4.30	99	0.49	0.71	1.57	2.69
DSMQ 14 (-)	99	0.83	0.55	-0.06	-0.12	99	0.80	0.62	0.16	-0.60
DSMQ 15 (-)	99	0.75	0.58	0.07	-0.51	99	0.79	0.66	0.25	-0.79
DSMQ 16	99	0.60	0.64	0.58	-0.66	99	0.52	0.61	0.74	-0.47
